# The Rate of and Factors Associated with Delivery by Caesarean Section among Women with Epilepsy: Time Trend in a Single-Centre Cohort in Mazovia, Poland

**DOI:** 10.3390/jcm11092622

**Published:** 2022-05-06

**Authors:** Beata Majkowska-Zwolińska, Joanna Jędrzejczak

**Affiliations:** 1 Epilepsy Diagnostic and Therapeutic Center, Foundation of Epileptology, 02-952 Warsaw, Poland; 2 Centre for Postgraduate Medical Education, Department of Neurology and Epileptology, 00-416 Warsaw, Poland; jjedrzejczak@cmkp.edu.pl

**Keywords:** women with epilepsy, pregnancy, caesarean delivery, seizures, antiseizure treatment

## Abstract

Data from literature suggest that the rate of caesarean section (CS) in women with epilepsy (WWE) is higher than in the general population. In Poland, there is neither a national registry nor another data set to access the outcome of pregnancy in WWE. Therefore, we address this gap by prospectively studying CS rates among 1021 WWE pregnancies at a single centre, their trends over time, and factors increasing the likelihood of the CS. To determine whether the diagnosis of epilepsy itself increased this likelihood, mixed models were used to analyse the contributions of specific variables, including the presence of seizures at different pregnancy-related timepoints. Over 20 years, the mean rate of CS in WWE was progressively growing and was higher than in the general population in Mazovia (47% vs. 32%). Generalized seizures in the third trimester increased the likelihood of CS with the highest odds (OR 4.4). The most frequent indication for a CS was obstetric (58.1%), followed by epilepsy-related (25.2%). Almost half of women who indicated epilepsy as the sole reason for CS had no seizure during pregnancy, and nearly 70% did not have generalized seizures. This suggests the overuse of epilepsy as an indication of CS and encourages defining more strict criteria.

## 1. Introduction

The incidence of caesarean section delivery (CS) in the general population is increasing worldwide, although CS is a major surgical procedure associated with short- and long-term health risks to both the mother and infant [1]. In addition, according to new research from the World Health Organization (WHO), those rising rates may suggest an increasing number of medically unnecessary procedures [2].

Epilepsy is one of the most common chronic neurological disorders during pregnancy [3,4,5,6]. Although pregnancy in women with epilepsy (WWE) may be considered a high-risk obstetrical condition, most WWE have uneventful pregnancies, have a relatively low risk of complications during labour and delivery, and can deliver vaginally (VD) [7]. However, increased CS, labour induction, and other obstetric interventions have been reported [8,9,10,11,12,13,14,15]. As all chronic conditions have been found to increase the likelihood of CS [16], epilepsy itself may play a role. Furthermore, the frequency of seizures may change and significantly affect the course of pregnancy and delivery [17,18]. In addition, women with active epilepsy undergo planned CS more frequently [15,19,20].

There is not much information on whether there is an increasing parallel trend over time of CS rates in WWE, likewise in the general population. However, most publications show that CS is performed more frequently in WWE than in the general population [9,14,19,21,22]. The reason for this is not apparent and probably involves multiple factors, but publications investigating these reasons are scarce.

The threat of seizures during delivery and their potential consequences may be the most important reason for concern for both WWE and clinicians and may influence their preference for CS. However, around half of WWE do not have seizures during their pregnancy [23,24,25,26]. Thus, it is unclear whether CS in WWE is performed because of seizures during pregnancy or because of the diagnosis of epilepsy itself, regardless of seizure occurrence.

In Poland, there is neither a national registry for WWE nor other data sets to access the outcome of pregnancy in WWE. In one study from Poland, half of 171 pregnancies among WWE were reported to be delivered by CS [27]. Therefore, with long-term observation of a large cohort of WWE, we address this gap by studying the CS rates among WWE, their trends over 20 years, and factors increasing the likelihood of CS.

To determine if the diagnosis of epilepsy itself is overused as an indication of CS for WWE, we studied the actual documented reasons for CS, which is a novel approach. In addition, we hope to provide useful information that may guide clinical practices at the intersection of neurology and obstetrics.

## 2. Materials and Methods

### 2.1. Study Design

Data on delivery mode were prospectively collected at the outpatient tertiary Epilepsy Centre in Warsaw (Mazovia County, Poland), along with other information on pregnancy in WWE that resulted in births between January 2000 and December 2019. Data were recorded throughout pregnancy and postpartum through a standardized form created in 2000 and stored in an electronic institutional database. It was initially aimed to assess the maternal and neonatal outcomes.

The inclusion criteria for this study were pregnant women with an established diagnosis of epilepsy who gave birth, with enrolment before 16 weeks of gestation, regular once per trimester clinical follow-up, postpartum period visit up to six months, and who were willing to participate in the study. The exclusion criteria were women with nonepileptic seizures or those unable to provide consent.

We included multiple pregnancies as women’s profiles differed enough to be considered independently as per age, antiseizure medications (ASM) use, parity, seizure occurrence, mode of delivery, and birth outcome.

Patients were informed of the study purpose and the anonymity of their data and were assured that medical treatment decisions were independent of their provision of informed consent or preference not to participate in the study. Written or oral informed consent was obtained from all participants. The study was approved by the local ethics committee (Centre of Postgraduate Medical Education, Warsaw, Poland, approval number 124/PB/2019).

### 2.2. Data Collection

Data for analysis included maternal demographic and obstetric information, namely epilepsy type, seizure type, its occurrence, use of antiseizure medications, maternal age at conception, gravidity, gestational week, presence of congenital malformation, and mode of delivery. Maternal baseline data and information on the occurrence of seizures during pregnancy were prospectively collected during visits by the physicians. Data on seizures occurring one year prior to pregnancy were collected retrospectively through medical records and clinical interviews. Seizures were classified under the new operational classification [27]. We analysed the presence of seizures of any type (focal and generalized) within one year prior to pregnancy and during the entire pregnancy. Given the potentially most significant impact of seizures in the third trimester on the decision on the mode of delivery, the presence of generalized seizures was studied separately in this period. For analysis purposes, generalized seizures included only focal-onset to bilateral tonic-clonic seizures and generalized-onset tonic-clonic seizures.

Data on ASM use during pregnancy were recorded in each trimester of pregnancy. ASM use was categorized as no drug use, monotherapy (if one ASM was used), and polytherapy (if two or more ASMs were used).

The primary outcome was the mode of delivery, and the secondary outcome was indications for CS. Generally, they are reported as either elective or emergent. Employing a different approach, our analysis presents the actual documented reason for the CS. Based on the birth medical records of the WWE, the indications for CS were divided into three categories: obstetric, epilepsy-related, and other indications. Epilepsy-related indications given by the obstetrician as an indication for caesarean section include a diagnosis of epilepsy per se, regardless of seizure occurrence.

The study’s endpoints were (1) associations between factors related to the use of CS in the WWE deliveries cohort and (2) differences in CS rates among WWE and in the general population of women in Mazovia. To calculate CS rates as a comparator, we used the number of deliveries (physiological and CS deliveries) in Mazovian County hospitals in the period 2000–2019. These data were available in publications of the Mazovia Centre of Public Health [28] and annual reports of the National Health Found [29]. This collection contains information on 1,108,053 deliveries among the entire Mazovia population.

### 2.3. Statistics

All calculations were performed in the R 3.6.0 statistical package (R Foundation for Statistical Computing, Vienna, Austria). The unit of analysis was total deliveries of WWE. Quantitative variables are presented as the mean and standard deviation, median and interquartile range (IQR) or range. For categorical variables, percentages were calculated for individual factors and the number of patients in each category. The Mann–Whitney U test was used to compare the values of numerical variables in the two groups. The two categorical variables were compared using Fisher’s exact test or the chi-square test. One-way mixed models with random intercepts for patients were used to identify the factors influencing the likelihood of CS. Based on these models, the values of the odds ratios (ORs) for CS were calculated. The ORs for individual models are presented as a forest plot. Correlations between year and the CS rate, as well as indications and the year of the study period, were investigated using a linear regression model. To identify differences in mean caesarean section rates according to data sources, the Kruskal–Wallis test and post hoc Dunn tests were applied.

## 3. Results

### 3.1. Study Sample

The total number of WWE pregnancies entered in the database between 2000 and 2019 was 1572. In total, 363 were lost to follow-up, and 184 were excluded due to miscarriages. Due to a small number, four forceps/vacuum deliveries were excluded. Finally, 1021 deliveries in 864 WWE were analysed. A total of 248 WWE entered the analysis more than once.

The average maternal age was 28.55 years, ranging between 17 and 45 years. Most WWE (64%) had focal epilepsy, and the rest had generalized epilepsy. The majority were primiparous (65.8%). A large majority of WWE (91.6%) gave birth on time between 37- and 42-weeks’ gestation, 8.1% gave birth prematurely before the 37th week, and 0.3% after the due date.

Data comprising the percentages of any type of seizure and generalized seizures occurring within one year prior to conception, during the entire pregnancy, and during the 3rd trimester in WWE who gave birth vaginally or by CS are presented in Table 1. Any type of seizure one year prior to conception occurred in 47% of WWE and 52% in the entire pregnancy. During pregnancy, 28.3% had a generalized seizure, with only 11% in the third trimester.

The numbers and percentages of pregnancies delivered vaginally and by CS according to epilepsy treatment at conception and in the 1st, 2nd, and 3rd trimesters of pregnancy are presented in Table 1. The majority of women were treated with ASM. The percentage of untreated varied, depending on the period of pregnancy, from 12.8% to 14.1%.

The detailed clinical description of the WWE pregnancies is presented in Table 1.

### 3.2. Comparison of CS vs. VD Deliveries in WWE

Over the study period, there were 500 (49%) CSs and 521 (51%) VD among the WWE. WWE who delivered by CS significantly more often than WWE who gave birth vaginally were older (*p* = 0.002) and had a lower gestational week at delivery (*p* < 0.001), had more seizures of any type, as well as generalized seizures in all trimesters (*p* < 0.001). The association between pre-pregnancy epileptic seizures and caesarean section was not shown to be statistically significant (*p* > 0.05).

In each trimester of pregnancy, monotherapy was the most commonly used treatment regimen in both the CS group and the WWE who gave birth vaginally. In all trimesters, a higher proportion of women treated (either with monotherapy or polytherapy), compared to untreated women, had a CS. However, at conception, such a relationship was found only for women treated with polytherapy. The detailed clinical description of the WWE pregnancies according to delivery mode is presented in Table 1.

### 3.3. CS Rate and Its Changes over Time in WWE and the Mazovia General Population

**Figure 1:**
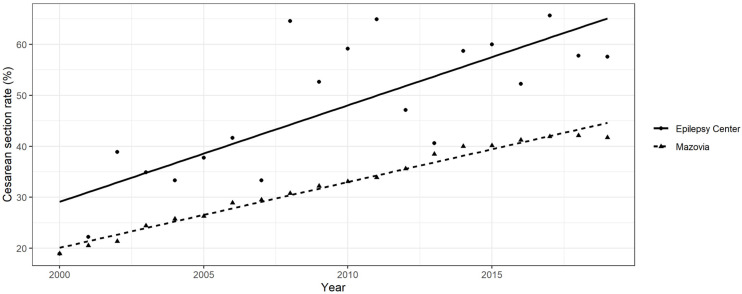
Linear regression of caesarean section rate by year for patients in the epilepsy centre (WWE) and the general population of Mazovia County.

Over the period 2000–2019, the mean CS rate for the epilepsy centre was 47.1 (SD 14.26; range 18.92–65.67) and was significantly higher than the mean CS rate for the Mazovia County population—32.35 (SD 7.7; range 18.95–42.09), *p* = 0.001. The annual CS rates for the epilepsy centre and the Mazovia population over the 20 years are shown in Figure 1. According to regression analysis, CS rates were significantly affected by both the year and source of the delivery data (WWE cohort and the general population of Mazovia). Among the WWE at the epilepsy centre, there was an increase in the CS rate from 18.92% to 57.58%, with a linear increase of 1.89% annually (95% confidence interval (CI): 1.15–2.63, *p* < 0.001), over the 20-year study period. In the Mazovia County population, the CS rate increased from 18.95% to 41.71%, with a linear increase of 1.28% annually (95% CI: 1.19–1.38, *p* < 0.001) in the years 2000–2019.

The number of deliveries in Mazovian County hospitals in the period 2000–2019 for analysis was obtained from publications of the Mazovia Centre of Public Health [28] and annual reports of the National Health Found [29].

### 3.4. Analysis of Factors Related to CS in WWE Deliveries

The effects of individual factors on the performance of CS described below (seizures, AES treatment, gestational week at delivery, parity, primiparity, twin pregnancies) were examined using one-way mixed-effects models. Individual tendencies for CS were considered for each patient (Table 2).

#### 3.4.1. Seizures

**Figure 2:**
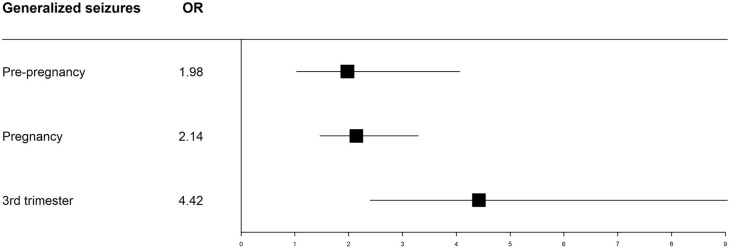
Likelihood of caesarean section according to the occurrence of generalized seizures pre-pregnancy, throughout the entire pregnancy, and in the 3rd trimester. Odds ratios (ORs) for caesarean section are presented as a forest plot with corresponded 95% confidence interval.

In the WWE, the relationship between any type of seizure pre-pregnancy and CS was not statistically significant. However, the odds of a CS were almost twice as high (1.97) in WWE who had generalized seizures one-year prior to pregnancy than in those without generalized seizures (Table 2). The occurrence of any type of seizure during pregnancy was significantly associated with CS (*p* < 0.001). Additionally, there was a progressive increase in the likelihood of a CS in cases of generalized seizures, with an almost 4.5-fold increase in the 3rd trimester (Figure 2). Only two patients had seizures during labour that motivated a CS delivery.

#### 3.4.2. Antiseizure Treatment

The likelihood of CS according to antiseizure treatment is presented as odds ratios and *p*-values in Table 2. Polytherapy at each point of observation (at conception and in trimesters 1, 2, and 3) progressively increased the likelihood of CS compared to no therapy. Monotherapy had a lower but more stable effect on the likelihood of CS than no therapy in each trimester, but the effect was not significant at conception.

#### 3.4.3. Gestational Week at Delivery

The mode of delivery was significantly correlated with the gestational week. The mean gestational week at delivery was significantly lower for WWE who underwent CS than for WWE who had a vaginal delivery (mean: 38.41 vs. 39.14 weeks, *p* < 0.001) (Table 1). The logistic mixed-effects regression analysis showed that the duration of pregnancy significantly influenced whether CS was performed (OR 0.78; *p* < 0.001). With each gestational week increase in pregnancy duration, the patient’s likelihood of undergoing a CS decreased by approximately 1.3 fold (Table 2).

#### 3.4.4. Parity

A mixed logistic model was used to analyse the probability of CS. In this model, it was assumed that the probability of pregnancy ending by CS is influenced by the number of pregnancies the patient has had. The individual probability of CS in each woman was also considered. According to the model, the number of pregnancies experienced by the patient does not affect the likelihood of CS.

#### 3.4.5. Twin Pregnancies

Analysis by Fisher’s exact test revealed that the mode of delivery was affected by twin pregnancy and that the relationship was significant (Table 1). The vast majority (85.7%) of twin pregnancies ended in CS. The twin pregnancy significantly affected the probability of CS (OR 4.1) (Table 2).

To summarize, seizures in the 3rd trimester and twin pregnancies were the most crucial among all factors increasing the likelihood of CS.

### 3.5. Indications for CS

The indications for CS were determined for 485 pregnancies out of 500 in the WWE cohort. There was more than one indication in 4.9% (24) of deliveries. The most frequent indications for CS were obstetric (282; 58.1%), followed by epilepsy-related (140; 28.9%) and other indications (85; 17.5%). The most common obstetric indications were prolonged and obstructed labour (30.5%) and foetal distress (19.1%). Among other indications, neurological ones were most common (36.5%). Detailed obstetric and other non-epilepsy-related indications for CS are listed in Table 3.

#### Seizure Occurrence during Pregnancy According to the Group of Indications for CS

Among 265 WWE for whom the decision to perform CS was based solely on obstetric indications, 136 (51.3%) had any type of seizure during pregnancy, and 27.7% (*n* = 73) had generalized seizures. In the group of patients with other indications for CS (70), the rates of seizure occurrence were similar (any type of seizure, 51.4% *n* = 36: generalized seizures, 27.1% *n* = 19). Among pregnancies in which an epilepsy-related reason was the only indication for CS (123), 54.9% (*n* = 67) had any type of seizure throughout the entire pregnancy, and 31.1% (38) had generalized seizures.

To summarise, the occurrence of any type of seizure, as well as generalized seizures during pregnancy regardless of the group of indications, were roughly comparable.

### 3.6. Relationship between CS Indication and Year of the Study Period from 2000–2019

To check whether the distribution of indications for caesarean section has changed over the 20 years, a linear regression model was used for each indication. There was no significant effect of year on CS for any of the indications: (obstetrical indication, OR—0.86; *p* = 0.170; epilepsy-related, OR—1.19; *p* = 0.139; other OR 2.71; *p* = 0.131). So, the tendency to perform a caesarean section for any three reasons (obstetric, seizure, other) has not changed over time.

## 4. Discussion

The results are based on the analysis of a single centre, but they can shed light on the application of CS in Poland, as our study comprises a large cohort of WWE over an extended observation period. This allowed us to address the gap of the CS rates among WWE, their trends over 20 years, and factors increasing the likelihood of the CS, as well as indications for CS in Poland.

Over the study period, the mean CS rate in our cohort was 47.1%. An increased rate of CS in WWE is common across worldwide studies [8,16,17,20,30,31,32] but not consistent [33,34,35]. Differences in methodology, group size, and study period in recent decades do not always allow a direct comparison. In our study, the CS rate was higher than the reported rates in other European countries (19.8%; [32]; 37.6% [18]) and Australia (39.2%) [16] in the last decade but lower than in Taiwan (54.5%) [17]. Our high rate may, to some extent, reflect the increasing percentage of CSs in the general population of Poland, leading to what is now one of the highest CS rates in Europe, exceeding 40% [29,36].

Over 20 years, the rate of CSs in the WWE at our centre has progressively increased, and at every point of observation it was higher than that in the general population in Mazovia County (32.3%). Our increasing trend is in line with a recent meta-analysis that indicated a global trend towards increasing CS rates among WWE over time, with significant geographical variability [37]. The exception is in a Norwegian study based on data from a national registry: although an increase in elective CSs in WWE was observed, there was no increase over time [11]. However, compared to other European countries, Norway has very low CS rates overall and among WWE [36].

Given that caesarean section is a medical procedure with potential adverse consequences, it is essential to establish whether such a high rate, increasing over time globally, is justified.

Our analysis showed that a number of factors were significantly associated with an increase in the likelihood of CS, such as seizures, twin pregnancy, ASM treatment, and a gestational week at delivery. In a similarly long observation of nearly 20 years, but based on a registry study, Vajda [16] similarly found twin pregnancies to be associated with an increase in the likelihood of CS, but not seizures or ASM treatment. However, the effect of mono or polytherapy is rarely assessed in the literature, with inconsistent results [16,17,20,38]. In our patients, polytherapy at every stage of pregnancy was an important factor affecting the likelihood of CS.

In most studies based on national registries, seizures are rarely considered, as it is not always possible to do so [16]. Notably, this single-centre analysis allowed us to consider the impact of various types of seizures throughout pregnancy and pre-pregnancy on the mode of delivery. This analysis highlights the significant impact of the type of seizure and the time of its occurrence on the decision to perform a CS. While the occurrence of any type of seizure one year prior to pregnancy did not significantly impact CS, the occurrence of pre-pregnancy generalized seizures almost doubled the likelihood of a CS. At each stage of pregnancy, the occurrence of any type of seizure, especially a generalized seizure, was associated with an even greater likelihood of CS, with the highest odds (4.5 times higher) in the third trimester. In a Brazilian study [38], CS in women with drug-resistant epilepsy was correlated with increased seizure frequency and poor seizure control; however, no information on seizure type or pregnancy stage was included. Therefore, our observation may be important in daily practice and emphasizes the need to optimize seizure control, especially for generalized seizures, in pregnancy.

Very few studies have addressed the reasons or indications for CSs in WWE [16,17,18]. Generally, they are reported as either elective or emergent. However, this approach in WWE does not seem to address clearly what is the relation of seizure occurrence or other factors related to epilepsy with elective CS. Therefore, we have attempted to assess the causes reported in the medical records as related to epilepsy, which allegedly suggests the presence of seizures.

Obstetric indications in our patients, which are the most common group of CS indications, accounting for nearly 60% of CSs, do not raise doubts in terms of why CS is the chosen delivery mode; however, when epilepsy-related factors are the only reason for a CS, one may wonder whether the frequent performance of CSs is justified. Epilepsy-related indications accounted for more than one-quarter of CS indications in our study. The incidence of any type of seizure, as well as generalized seizures alone, was only slightly higher among women with epilepsy-related indications than among those with obstetric and other indications. When the former subgroup was analysed in more detail, it was found that almost half of the women had no seizures throughout their entire pregnancy and that nearly 70% did not have generalized seizures. This finding suggests that CS delivery may not have been fully justified.

Many obstetricians and neurologists, fearing seizures during delivery, choose preventive CS, thereby maintaining the notion that epilepsy in pregnancy is an indication for CS, regardless of existing recommendations. International guidelines recommend vaginal delivery for WWE [9,38,39,40,41]; however, elective CS may be considered for those with seizures during labour or in cases of a lack of cooperation [42] and in a small proportion of WWE with significant deterioration in terms of seizures that are recurrent and prolonged throughout pregnancy [39,41]. Only 16.2% of women with CS deliveries had generalized seizures in the third trimester in our group. Moreover, only two of them had generalized seizures during labour ending in CS, which is in line with the observation that seizures do not commonly occur during labour [3,22].

The tendency to perform CS for any three reasons (obstetric, seizure, other) has not changed over the 20-year study period. It seems that the belief that epilepsy is an indication for CS is well established and has persisted over time among obstetricians, neurologists, and patients, which is in line with recent observations of an Australian registry-based study over 19 years [16]. Thus, education is needed. Knowledge of pregnancy-related issues in Poland regarding the WWE of reproductive age is still unsatisfactory. Slightly less than 40% of women believe that epilepsy alone is not an indication for CS [43,44].

Moreover, in addition to the medical reasons for higher CS rates among WWE, proposed non-nonmedical reasons include causes not strictly related to medical needs, such as policies promoting subsequent CS and patient/obstetrician-related factors (e.g., maternal request, tocophobia, fear of malpractice accusations), in the general population of women and obstetricians [45]. These reasons may also apply to WWE. As tocophobia is becoming a growing obstetric problem in the general population [46], further research is needed to determine how this phenomenon may play an even greater role in WWE.

Our study has some limitations. A single-centre study located in Mazovia, the largest county in Poland allowed us to accumulate detailed information on a large cohort of WWE; however, it might not be representative of Poland. As a reference centre, we are likely to have more patients with more severe epilepsy under our care. However, there is no systemic care for pregnant WWE in Poland, so there is no other source of structured data. Moreover, our group comprised quite a considerable number of WWE with more than one pregnancy. However, they differed substantially for several factors such as age, parity, AED profile, seizure frequency, pregnancy, birth outcomes, etc., so they could be considered independently.

## 5. Conclusions

From 2000 to 2019, the rates of CS among WWE have progressively increased, and the CS rate in WWE is higher than that in the general population in Mazovia, Poland. Although the presence of generalized seizures, especially in the third trimester of pregnancy, substantially increases the odds of CS, the actual incidence of seizures is relatively low. It does not justify such frequent use of CS, including in women whose only reason for CS is an epilepsy-related indication. These findings fill gaps in our understanding of this problem in Poland and have potential wider application at the interface of neurology and obstetrics. The results may encourage more careful monitoring of pregnancies among WWE, treatment optimization, and more well-defined indications for CS.

## Figures and Tables

**Table 1:** General clinical descriptive of WWE pregnancies and according to the mode of delivery (*n* = 1021).

				Vaginal Delivery				Caesarean Section				*p*
Variable				*n*	Mean		SD	*n*	Mean		SD	
Maternal age at delivery (years)				521	28.04		4.91	500	29.02		4.51	**0.002 ^1^**
Gestation week				521	39.14		1.95	500	38.41		2.14	**<0.001 ^1^**
		Total deliveries		*n*		%		*n*		%		
		*n*	%									
Treatment at conception	No ASMs	144	14.1	85		16.3		59		11.8		0.057 **0.019**
	Monotherapy	711	69.6	360		69.1		351		70.2		
	polytherapy	166	16.3	76		14.6		90		18		
Treatment in 1st trimester	No ASMs	131	12.8	80		15.4		51		10.2		**0.019** **0.008**
	Monotherapy	724	70.9	365		70.1		359		71.8		
	polytherapy	166	16.3	76		14.6		90		18		
Treatment in 2nd trimester	No ASMs	142	13.9	86		16.5		56		11.2		**0.02** **0.004**
	Monotherapy	724	70.9	366		70.2		358		71.6		
	polytherapy	155	15.2	69		13.2		86		17.2		
Treatment in 3rd trimester	No ASMs	131	12.9	79		15.2		52		10.4		**0.042** **0.003**
	Monotherapy	728	71.3	373		71.6		355		71.2		
	polytherapy	161	15.8	69		13.2		92		18.4		
Any type of seizures 1 year prior to conception	No	165	53.0	89		56		76		50		0.273
	Yes	146	47.0	70		44		76		50		
Generalized seizures 1 year prior to conception	No	498	88.0	262		90.7		236		85.2		**0.044**
	Yes	68	12.0	27		9.3		41		14.8		
Any type of seizures during pregnancy	No	488	48.0	278		53.5		210		42		**<0.001**
	Yes	532	52.0	242		46.5		290		58		
Generalized seizures during pregnancy	No	731	71.7	401		77.1		330		66		**<0.001**
	Yes	289	28.3	119		22.9		170		34		
Generalized seizures in the 3rd trimester	No	787	89.0	430		93.9		357		83.8		**<0.001**
	Yes	97	11.0	28		6.1		69		16.2		
Twin pregnancy	No	1007	98.6	519		51.5		488		48.5		**0.006 ^2^**
	Yes	14	1.4	2		14.3		12		85.7		
Malformations	No	948	92.9	485		94.5		463		91.3		0.1314 ^3^
	Major	24	2.4	9		1.8		15		3		
	Minor	48	4.7	19		3.7		29		5.7		

^1^ Mann–Whitney U test, significance bolded. ^2^ Fisher’s exact test. ^3^ Chi-square test. ASM—antiseizure medication.

**Table 2:** Factors associated with caesarean section.

Variable	Odds Ratio	*p*-Value
Generalized seizures in the 3rd trimester	**4.42**	**<0.001**
Twin pregnancy	**4.10**	**0.022**
Polytherapy in the 3rd trimester	**2.71**	**0.003**
Polytherapy in the 2nd trimester	**2.55**	**0.004**
Polytherapy in the 1st trimester	**2.38**	**0.008**
Generalized seizures any time during pregnancy	**2.13**	**<0.001**
Polytherapy at conception	**2.08**	**0.019**
Generalized seizures 1 year prior to pregnancy	**1.97**	**0.044**
Monotherapy in the 1st trimester	**1.85**	**0.019**
Any seizure any time during pregnancy	**1.82**	**<0.001**
Monotherapy in the 2nd trimester	**1.81**	**0.020**
Monotherapy in the 3rd trimester	**1.70**	**0.042**
Monotherapy at conception	1.60	0.057
Seizures 1 year prior to pregnancy	1.32	0.273
Parity	0.96	0.821
Primiparity	0.89	0.703
Gestational week	0.78 *	**<0.001**

* 1/0.78 = 1.27, significance bolded. Coefficients of one-way mixed-effects models explaining the occurrence of caesarean section; The odds ratios (OR) and *p*-values for each prepared model ranked from most to least common.

**Table 3:** Summary of obstetric and other than epilepsy-related indications for caesarean section.

Indications for Caesarean Section					
Obstetric			Other		
	*n*	%		*n*	%
Prolonged and obstructed labour	86	30.5	Neurological: cerebral palsy, aneurysm, tumour, brain/brainstem cavernoma, hemiparesis, haemorrhage Arnold-Chiari syndrome	31	36.5
Foetal distress	54	19.1	Orthopaedic: hip dislocation/joint dysplasia/instability, spine defect	17	20
Abnormal positioning	36	12.8	Ophthalmic: myopia, retinal detachment	16	18.8
Cephalic-pelvic disproportion	27	9.6	Internal/metabolic	8	9.4
Previous caesarean section	25	8.9	Psychiatric/mental state: psychosis, psychogenic seizures, mental impairment, uncooperativeness	8	9.4
Urogenital tract infections, uterine defects, in vitro fertilization	14	5.0	Cardiac: heart defect, circulatory failure, hypertension	5	5.9
Placenta/amniotic fluid disorder	12	4.3			
Preeclampsia/eclampsia	11	3.9			
Multiparity	10	3.5			
Foetal malformation/stillbirth	4	1.4			
Abnormal intrauterine growth	3	1.0			
Total	282	100		85	100

Indications ranked from the most common to the least frequent.

## Data Availability

Data are stored in an electronic institutional database at Epilepsy Diagnostic and Therapeutic Center, Foundation of Epileptology, Warsaw, Poland.
